# Surface Enhancement of Titanium Ti-3Al-2.5V Through Laser Remelting Process—A Material Analysis

**DOI:** 10.3390/mi15121526

**Published:** 2024-12-22

**Authors:** Esmaeil Ghadiri Zahrani, Babak Soltani, Bahman Azarhoushang

**Affiliations:** 1 Institute for Advanced Manufacturing (KSF), Furtwangen University, 78532 Tuttlingen, Germany; esmaeil.ghadiri.zahrani@hs-furtwangen.de (E.G.Z.); babak.soltani2001@gmail.com (B.S.); 2 Department of Microsystems Engineering (IMTEK), University of Freiburg, 79110 Freiburg, Germany

**Keywords:** titanium, laser remelting, phase transformation, microhardness

## Abstract

This study evaluates the effects of laser parameters on the surface remelting of the Ti-3Al-2.5V alloy. A ms-laser equipped with a coaxial gas-pressure head integrated into a Swiss-type turning machine is used for the laser remelting process of cylindrical parts. The influence of different pulse frequencies, as well as varying intensities, is investigated. The results reveal that surface micro-cracks can be eliminated through laser remelting. Increasing the input laser intensity also increases the size of the melting pool. A similar effect is observed with higher pulse frequencies. The metallurgical microstructure and the size of the heat-affected zone of the remelted surface at different input laser energy levels are also examined. The results indicate that input laser energy influences phase transformation in the metallurgical microstructure, which correspondingly results in variations in micro-hardness within the heat-affected zone. The variations in laser fluence lead to a surface hardness improvement of approximately 15%.

## 1. Introduction

From a tribological perspective, the functionality and reliability of an in-service surface are predominantly influenced by its surface roughness [1]. For certain applications, such as jewelry and marking, the visual impression is of utmost importance. To achieve the desired surface topography, post-processing techniques—primarily well-established conventional grinding and polishing processes—have historically been employed. However, these finishing processes become extremely challenging when free-form surfaces are involved or when machine kinematics reach their limits.

The laser polishing technique has recently been introduced as an alternative method to achieve surface roughness in nanometer range [2]. This method involves remelting a thin layer of material, allowing it to redistribute from peaks to valleys during solidification. As a result, surface irregularities are smoothed, leading to improved surface roughness [3]. The intensity distribution and pulse duration are the most critical parameters during laser polishing [4]. Depending on the laser pulse duration, two categories—macro-polishing and micro-polishing—can be defined [2]. In macro-polishing, continuous wave (CW) laser sources are typically used, while micro-polishing employs pulsed lasers, often with pulse durations in the microsecond (µs) and nanosecond (ns) ranges. Notably, for ns pulses, ablation rather than remelting is expected [5]. In recent studies, the focus has shifted to utilizing this process for finishing or functionalizing titanium parts for bio-implant applications [6,7,8,9]. The application of an inert gas during laser polishing of titanium enhances the microhardness of the surface layer and reduces surface roughness [6]. For titanium additive-manufactured parts, an 85% reduction in initial surface roughness could be achieved [10].

The challenge in processing titanium alloys lies in their unique combination of high strength—maintained at operating temperatures up to 400 °C—and low density [4]. This makes controlling the melt challenging, significantly impacting the final surface topography. Moreover, for titanium, it is essential to protect the surface from oxidation and crack initiation [11]. To address this, a vacuum atmosphere has been utilized in some cases [12], although this approach can restrict the working space and increase process costs. An alternative to the vacuum technique is the use of a protective medium, such as an inert gas. The presence of an assist gas ensures more uniform roughness reduction while minimizing the risk of molten droplet formation and oxidation [13,14]. Compared to the vacuum method, using an inert gas enables a process that is nearly size-independent, easier to automate, and more cost-effective. However, this approach comes with its own challenges. In laser remelting of Ti alloys, depending on the medium used for laser remelting, a passive film (Ti_2_O_3_ & TiO_2_) may form, resulting in improved corrosion resistance due to the homogenization of the composition in the remelting zone [15]. Alongside issues related to the material characterization, the control of the laser beam is also very important. Regardless of the beam acceleration and deceleration, another significant challenge is the “Laser On/Off delay” which arises from the synchronization between the laser power and the beam delivery system. This delay creates a transient zone, leading to variations in surface topography [16].

In summary, the efficiency of the laser polishing process depends on several factors, including laser parameters, material properties, and the laser beam delivery system. A deeper understanding of material behavior would therefore be highly beneficial. In this study, single laser spots were generated on the workpiece circumference using a high-power fiber laser. The alloy Ti-3Al-2.5V was selected as the material, and N_2_ was used as an inert gas to protect the surface. The alloy Ti-3Al-2.5V components, manufactured through the extrusion process, can exhibit surface defects such as micro-cracks. In this paper, the laser remelting process is employed to eliminate micro-cracks and evaluate the final surface roughness. While most studies on laser remelting focus on widely used titanium alloys like Ti-6Al-4V, the alloy Ti-3Al-2.5V has been less frequently studied. The facility used in this study provides a co-axial high-pressure inert gas to protect the remelting zone. The size of the resulting melt pool was analyzed, and a metallurgical investigation was conducted.

## 2. Experimental Details

**Figure 1:**
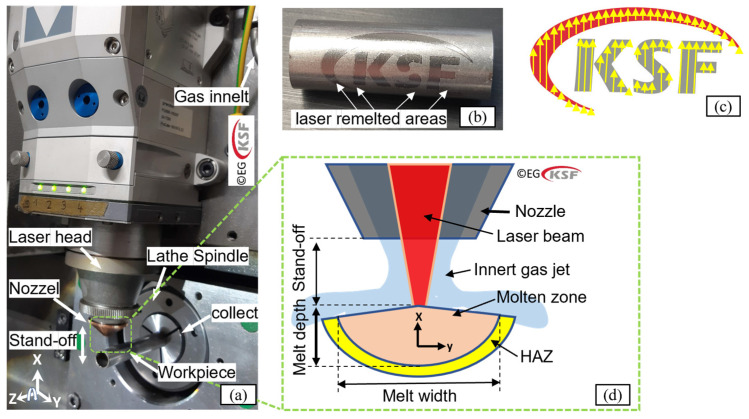
(**a**) Implement a fiber laser head into a CNC lathe, (**b**) Example of laser remelting, (**c**) Selected scanning strategy, and (**d**) Schematic of laser remelting under protective gas.

A fiber laser system (Fanuc FF3000i-A, Japan) was integrated into a CNC lathe (Maier MK36LASER, Wehingen, Germany). Figure 1a shows the laser head implemented into the machine. The laser head is positioned above the workpiece surface so that, during the laser irradiation, the workpiece rotates and simultaneously moves forward in the z-direction, depending on the scanning trajectories. As an example, Figure 1c shows the laser path (yellow arrays), which finally resulted in a polished contour of the KSF logo.

During irradiation, coaxial pressurized nitrogen gas is simultaneously blown out through the nozzle and acts upon the remelting spot (Figure 1d). A molten front is continuously formed (corresponding to each incident pulse) and then, protected by the coaxial gas targets as it targets the melting pool. This melt formation and melt displacement process continues until a desired remelted surface on the workpiece is achieved.

The workpiece, with a diameter of 20 mm, was made of Ti-3Al-2.5V. Different screening tests were conducted to determine the parameter range reported in this study. Three main criteria were selected: (1) no cutting, (2) no excessive thermal oxidation, and (3) no excessive melt displacement caused by high-pressure gas. Table 1 provides information on laser input parameters. The stand-off distance and nozzle diameter were kept constant throughout the experiments, and each test was repeated five times. During the experiments, the scanning speed (*V_L_*) and frequency (*F_L_*) were selected based on the concept that no overlap occurs between two sequential pulses. The beam spot (d) was always positioned on the surface for all tests. A confocal microscope (NanoFocus Mobile µsurf, Germany) was used to measure the surface roughness.

The energy intensity (*E_I_*) as defined in Equation (1), is inversely proportional to the scanning speed [3]:

(1) $$E_{I}=P_{L}/\left(V_{L}\times F_{L}\times d\right)$$

where *P_L_* is the laser power (W), d is the diameter of the beam (120 µm), and *V_L_* is the beam scanning speed (160 mm/s).

## 3. Result

**Figure 2:**
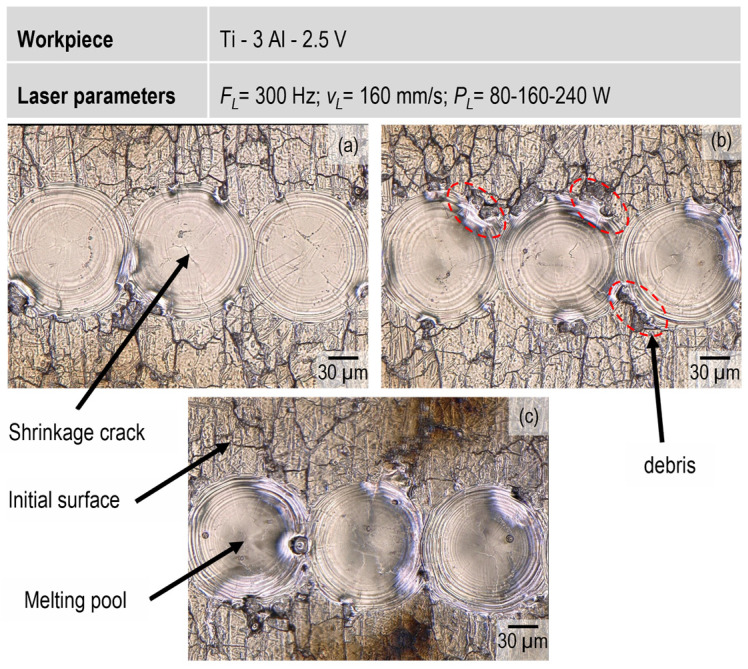
Elimination of surface cracks by laser remelting (**a**) 80 W, (**b**) 160 W, and (**c**) 240 W.

Figure 2 shows that every single incident pulse leads to a melting pool on the top surface of the workpiece. The dimensions and quality of the melt pool in the top layer may vary depending on the input energy level and pulse frequency. As illustrated in Figure 2, increasing the laser power from 80 to 240 W slightly enlarges the diameter of the melting pool and intensifies its surface morphology. However, increasing the laser frequency from 300 to 500 Hz, caused no considerable change in the melting pool shape. In all samples, shrinkage cracks were observed at the center of the melt pool. Two reasons could explain this issue. First, the distribution of the laser energy within the melt pool is not uniform due to the Gaussian distribution of the laser beam, causing the maximum input energy to be concentrated at the center. Second, the heating and cooling rates differ between the center and the edges of the melt pool. Consequently, a uniform distribution of thermal stresses is not achieved, leading to shrinkage cracks during melt solidification.

Regarding the debris formed at the edge of the melted zone (Figure 2), they could be identified as melt slags. As the laser intensity increases, the surface experiences significantly greater thermal damage along with the formation of slags. Typically, a layer of TiO_2_ forms on the surface of the bulk material [17,18]. During the localized remelting of titanium by laser irradiation, the oxide layer generally remains solid due to its higher melting point (approximately 1843 °C) compared to the base metal (1668 °C) or may partially melt under extreme conditions. Since titanium oxide has a lower density than titanium, it rises to the surface of the molten metal, forming a slag layer [19]. Due to the turbulence in the melt pool, the slags are pushed to the edges of the melting zone and solidified there, appearing as debris along the edges of the melt pool.

**Figure 3:**
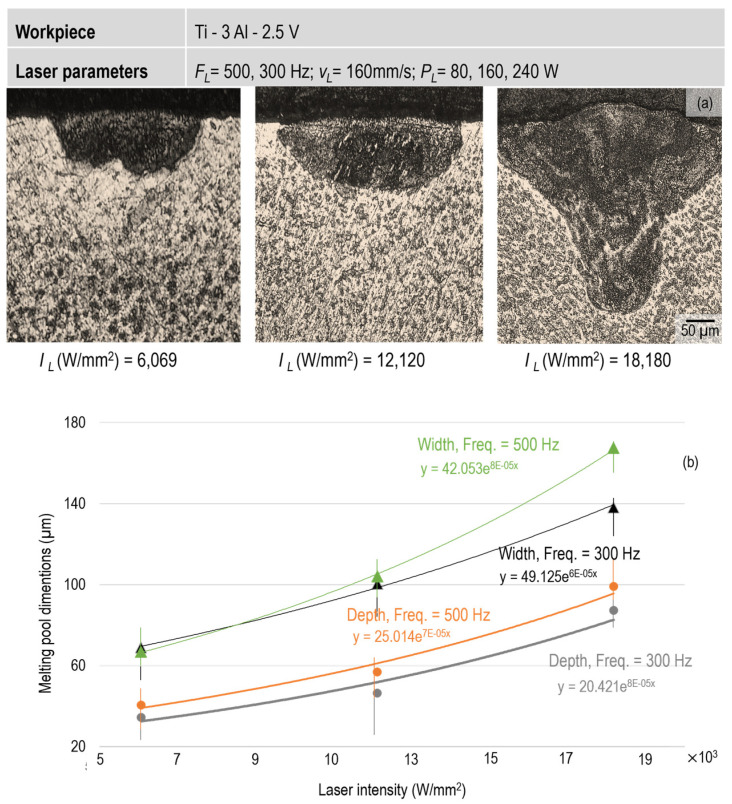
Effect of laser input intensity on the size of melting pool: (**a**) cross sections and (**b**) melting pool dimensions corresponding to different laser intensities.

Some cross sections of the laser-processed samples were prepared to investigate the depth and width of the resulting melting pool. For each test, three samples were diametrically cut and polished, followed by etching with Kroll solution. The melt pool dimensions were then measured. The experiments were conducted with laser intensities ranging from 6000 to 18,000 W/mm^2^ and frequencies of 300 and 500 Hz. Figure 3 shows the sample cross sections for the frequency of 500 Hz. After etching, the border between the solidified melting pool and bulk material is clearly distinguishable. Specifically, the melting pool exhibits a semi-circle shape for the intensities between 6000 and 12,000 W/mm^2^. This behavior arises from the energy distribution of the Gaussian laser beam, which follows an exponential energy decay from its center. At a laser intensity of 18,000 W/mm^2^ the shape of the melting pool transitions to a pine-like form. This occurs because an increased number of pulses leads to greater heat accumulation at the melt pool center, resulting in an increase in its depth.

Since the irradiated area under the beam spot experiences a high temperature and undergoes rapid thermal exchange with the surroundings within a short time, a steep temperature gradient is expected. This gradient causes a turbulent displacement of molten material inside the melt pool. Therefore, the upper layer of the melt pool during and after solidification, develops a wrinkled surface. As the laser input energy increases, these wrinkles become more pronounced.

Figure 3b shows the variation of melting pool dimension regarding the laser intensity and frequency. As the intensity increases, the melt pool expands. Similarly, at the same laser intensity values, an increase in frequency results in a proportional enlargement of both the melt width and depth. The best trends fitted for the parameter range and data studied here follow an exponential equation ($y=ae^{x}$), where *x* represents the laser intensity and melting pool dimension, respectively. Within the parameter range used in this study, it is evident that the melt pool expands as the laser frequency increases. Comparing different melting widths indicates that the b-value in the equation increases as the laser frequency rises. This suggests that a direct correlation exists between the laser off-time and melt penetration depth. In other words, when the rest time between two consecutive pulses decreases (due to a higher frequency), more energy is transferred into the irradiated material. This additional energy from subsequent pulses is largely absorbed by the melt pool. This leads to a significant increase in the melt temperature, particularly at the center of the melt pool. It is important to note that the rate of heat dissipation within the bulk material is inherently limited. Therefore, when higher-frequency pulses are applied to the melt pool, the molten material becomes more prone to movement, resulting in the formation of numerous surface wrinkles. Comparing the melt pool dimensions at F_L_ = 500 Hz reveals that the slope of the fitted function for the melt width is steeper than that for the melt depth. This indicates that the melt width compared to the melt depth is more sensible to the laser intensity. This can be attributed to the fact that a considerable amount of laser energy is absorbed by the upper layers of the melt pool, resulting in considerably higher temperatures at the top compared to the bottom. The minimum temperature within the melt pool corresponds to the melting temperature, while the maximum can exceed the boiling point, provided that the peak intensity is maintained long enough to overcome the atmosphere pressure. No material removal occurred within the parameter range used in this study. In laser processing, material removal typically occurs either through direct evaporation from the boiling melt pool or by using a pressurized gas to expel molten material from the melt pool. In this study, a pressurized gas (N_2_) at 3 bar was used, not to expel the molten material but to protect the surface from oxidation. The examination of the laser-remelted surface confirmed that no material removal took place here. The laser nozzle used in the experiments (Figure 1) did have an inner diameter of 5 mm, which prevented any mechanical shock from the N_2_ gas on the melt pool; ensuring no melt displacement occurred. Meanwhile, with this gas pressure applied, no evaporation was expected. According to Equation (2), applying the pressurized gas at 3 bar and considering the latent heat of 421 kJ/mol for titanium, the boiling point increased by 264 °C. This allowed for successful laser remelting without material removal.

(2) $$T_{B}={\left(\frac{1}{T_{0}}-\frac{RLn\frac{P}{P_{0}}}{∆H_{vap}}\right)}^{-1}$$

$∆H_{vap}$ = 421 kJ/mol, latent heat of vaporization

*R* = 8.314 J.K^−1^.mol^−1^, Gas constant

$\frac{P}{P_{0}}$: Vapor pressure to the atmosphere pressure at the sea level

*T_B_*: Boiling point (K)

*T*_0_: Boiling point at the sea level, 3560 K

It is important to note that the temperature at the edge of the melt pool should approximately equal the lower limit of the material’s melting temperature. Consequently, the temperature in the region near the molten pool and bulk material must lie between the melting point and the phase transformation temperature of the material in its solid state. The alloy used in this research is a two-phase titanium alloy, consisting of a background phase of β with an HCP structure and an α phase with a BCC lattice.

The α phase begins to transform into the β phase at a temperature of 450 °C to 650 °C. For the α phase to precipitate when the material is cooled from the phase transition temperature, the cooling rate must be slower than 0.1 °C/s [16], allowing the α phase to separate from the β phase and form a distinct phase. This cooling rate can only be achieved in aging heat treatment and within heat treatment furnaces.

The thermal conditions governing the laser process are similar to those of a welding process, characterized by high cooling rates and the formation of semi-equilibrium and non-equilibrium phases.

The heat-affected zone (HAZ) adjacent to the melt pool is expected in this situation. The formation of the HAZ zone not only alters the material’s microstructure but also affects mechanical properties. To investigate the HAZ, cross sections were prepared from the laser-remelted samples. Five samples were prepared from each experiment. The cross sections were first polished and then etched. Kroll’s solution with an HF 5% Vol. concentration was used at room temperature. Each sample was etched for ten seconds. The microstructure was then recorded under a light microscope (Keyence VHX5000, Japan).

**Figure 4:**
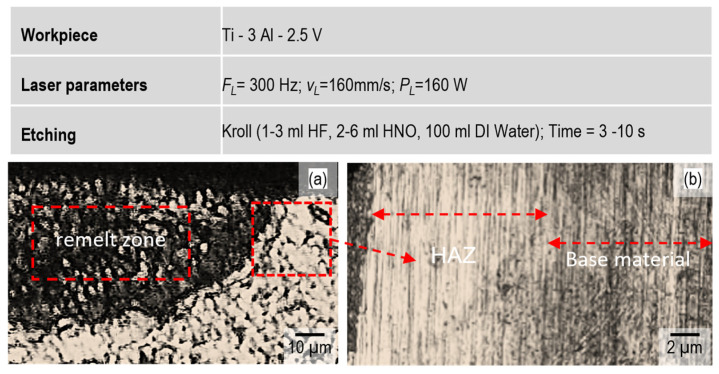
Investigation of (**a**) remelted zone, (**b**) HAZ and base material.

**Figure 5:**
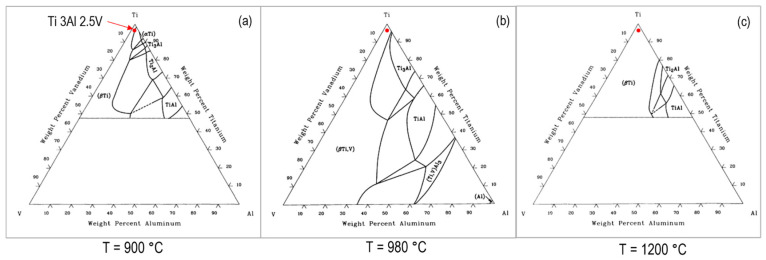
Titanium phase diagram at different temperatures [20]. (**a**) 900 °C, (**b**) 980 °C and (**c**) 1200 °C.

Figure 4a shows the cross-sectional microstructure of the laser-processed samples. Figure 4b shows the microstructure changes in the HAZ caused after laser remelting. In this region, the material was heated to a temperature between 450 °C and 650 °C, significantly lower than the solidus temperature (approximately 1650 °C). According to the phase diagram (Figure 5), at temperatures above 980 °C, the entire α phase dissolves into the β phase. In Figure 5, the location of the workpiece alloy is shown with a red dot. Due to non-equilibrium cooling, the α phase does not precipitate. Instead, a non-equilibrium phase with an HCP structure similar to the β phase dominates the altered region. This phase is also detected within the melt pool, as shown in Figure 4b.

**Figure 6:**
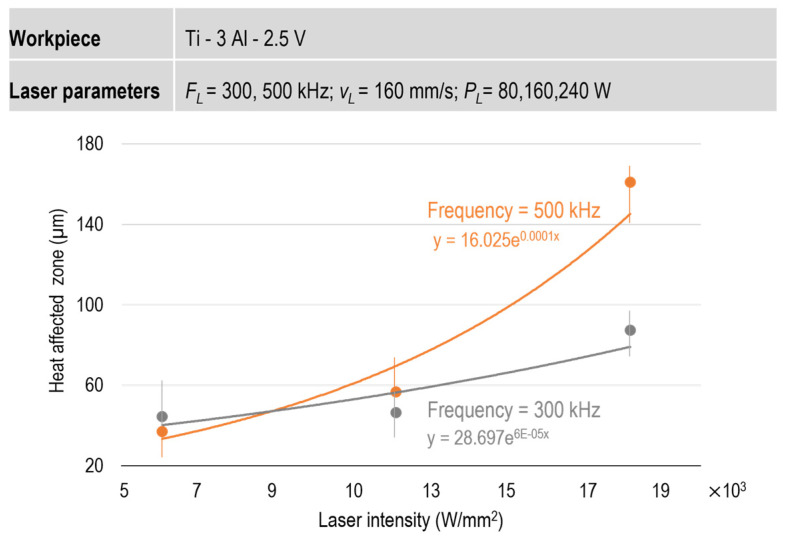
Effect of input laser intensity on the size of HAZ.

The width of the HAZ at different input laser intensities was measured for all five samples, and the results are summarized in Figure 6. As shown, the width of the HAZ increases with rising laser intensity. However, the sensitivity of the HAZ width is greater for the tests conducted at a laser frequency of 500 Hz compared to those at 300 Hz.

Comparing the thermal properties of the α-phase (which poses a BCC lattice) with non-equilibrium β-phases (which poses an HCP lattice) shows that as the β-phase increases, the thermal conductivity decreases from 17 to 7.5 W/m.K [21,22].

During the heating and phase transformation in the HAZ, the thermal conductivity in this region decreases by 50%. This reduction in thermal conductivity limits the dissipation of laser thermal energy within the workpiece. As the width of the HAZ increases, the transfer of heat from the melt pool to the surroundings further decreases. Consequently, the temperature of the melt pool rises, leading to an increase in the size of the HAZ.

In the HAZ region, the high cooling rate leads to the formation of non-equilibrium β’or α’ phases. The presence of these phases increases the hardness of the sample [21].

The results of the microhardness measurements in the laser-remelted area indicate that the hardness of the workpiece increases with the laser intensity.

During the phase transformation process, the initial stable phase must first transform into the β phase. This transformation requires maintaining the temperature at 900 °C or higher (Figure 5). At this temperature, all deposited α phase converts to β phase. This phase change is an equilibrium process, influenced by the penetration of aluminum atoms into the β phase and the rate of spontaneous penetration to convert the BCC structure to HCP.

The time required for the phase transformation controls the rate at which the transformation progresses.

The β phase produced during cooling transitions into a non-equilibrium phase. For this phase change to occur, the cooling rate must be comparable to that of a water quench, enabling the hardening process.

**Figure 7:**
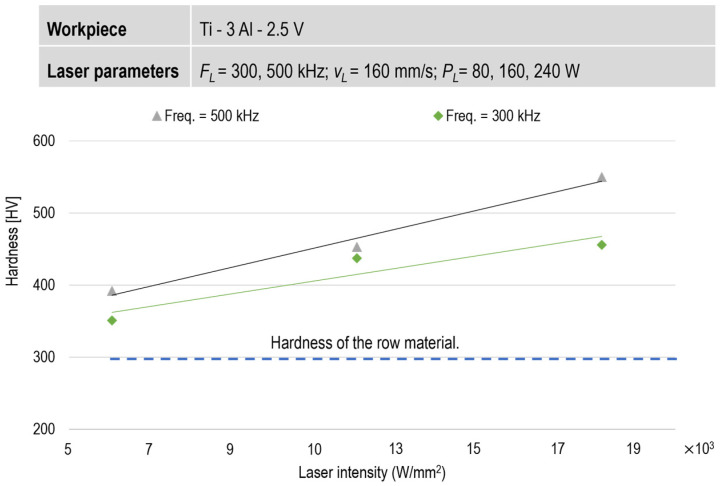
Effect of laser intensity on microhardness in the HAZ.

The percentage of β phase during the equilibrium phase transformation and the cooling rate determine the mechanical properties of the material after quenching. In the laser remelting process, increasing the laser intensity and frequency raises the laser process temperature and the thermal gradient in the HAZ. Consequently, the hardness of the remelted area also increases (Figure 7).

Another important factor in increasing microhardness is the effect of titanium nitride (TiN). During the laser remelting process of titanium alloy, using N_2_ as a protective gas may lead to interactions between the gas and the alloy elements. The extent and nature of this reaction depend on various factors, including the melt temperature and exposure time [23,24]. Nitrogen can dissolve in molten titanium, and its solubility increases at higher temperatures. This can alter the alloy’s lattice parameters, grain size, and grain number, as well as its mechanical properties [25]. At high temperatures, titanium exhibits a strong affinity for nitrogen, resulting in the formation of titanium nitride (TiN). The formation of TiN can lead to hard and brittle phases in the alloy, which may significantly increase its microhardness [24].

To achieve better surface quality, laser frequency and intensity should be limited. Under these conditions, both surface quality and hardness could be improved by at least 15%, with the hardness increasing from 300 HV to more than 350 HV. Additionally, the roughness of the remelted surface decreases from Ra = 1.5 µm to 1 µm.

Changing the laser frequency from 500 to 300 Hz decreases the surface roughness by 50%. At a frequency of 300 Hz, increasing the laser intensity to the maximum value results in a better surface quality compared to F_L_ = 500 Hz. However, at this frequency, the hardness of the remelted surface increases by 50%. Therefore, lower laser frequency combined with maximum laser intensity could be suggested to achieve the optimal balance between surface roughness and hardness.

**Figure 8:**
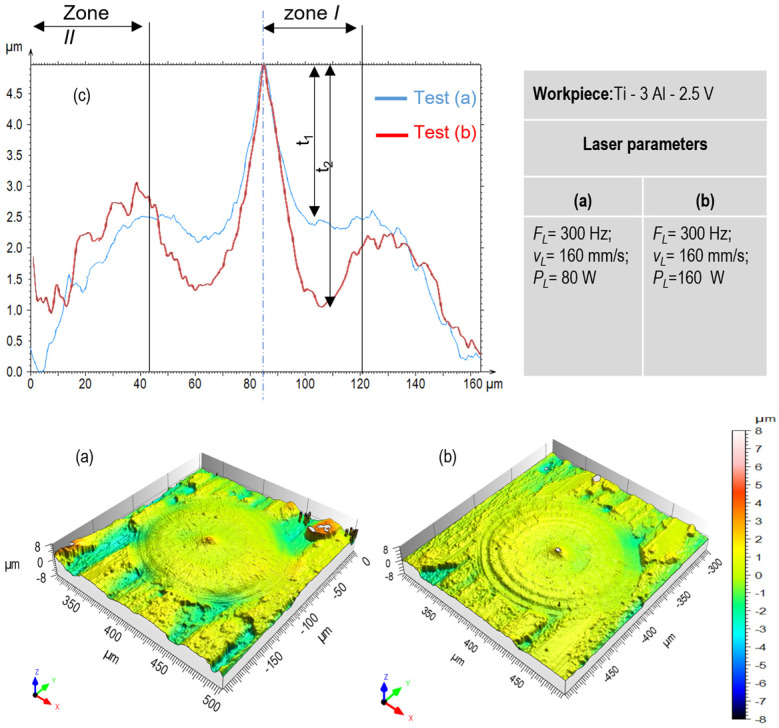
Evaluation of surface morphology after single pulse irradiation: (**a**) 80 W and (**b**) 160 W. (**c**) comparison of roughness profiles.

Looking closely at the melt morphology of a single pulse, some melting ripples at the melt pool borders are distinguishable (Figure 8a,b). Similarly, in the middle, a melt knurl affects the final quality of the remelted surface. As the confocal images show, the size of the melt ripples is approximately 1.5 µm. Figure 8c compares the surface topography obtained for the average powers of 80 W and 160 W across two distinct zones. Zone I is located near the center of the melting pool, whereas zone II is positioned around its edges. In zone I, the average power of 80 W (blue curve) results in a relatively smoother and less deep profile compared to 160 W (red curve), which exhibits higher amplitude and greater surface irregularities. This suggests a potentially rougher surface or increased melt displacement at the higher laser power. Comparing the values of t_1_ and t_2_, as marked in zone I, indicates deeper ripples when an average power of 160 W is applied. These observations align with the results in Figure 3, which show that the melting pool increases as the laser input density rises. In zone II, the difference between the two profiles is less pronounced because the laser intensity diminishes at the border of the melting pool.

**Figure 9:**
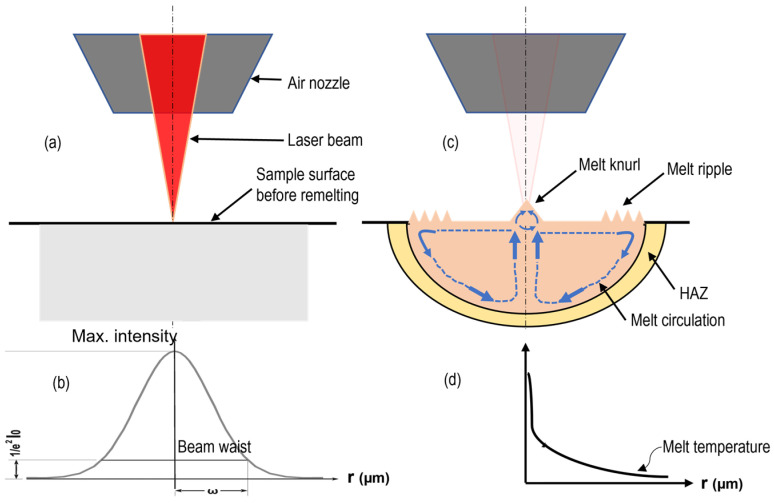
Formation of melt ripples: (**a**) process schematic, (**b**) laser intensity distribution, (**c**) melt ripple formation, and (**d**) distribution of melt temperature.

The laser used in this study has a Gaussian distribution, with the maximum intensity at the beam center that exponentially decreases to 13% of its maximum intensity at the edges (Figure 9b). Due to the maximal intensity at the beam center, the highest resultant temperature occurs at this point, corresponding to the melt pool center (Figure 9d). As subsequent pulses reach the melting pool surface, they cause localized thermal expansion at the upper layer of the melt pool. This thermal expansion of the melt, along with a reduction in melt viscosity, creates a temperature gradient and turbulence within the melt pool, as shown in Figure 9c. The turbulent circulation of the melt results in a rippled surface, particularly near the furthest areas of the melting pool adjacent to the bulk material. This process continues until no further pulses target the surface, after which rapid solidification occurs, forming the melt ripples.

## 4. Conclusions

This study investigates the laser remelting of titanium alloy using an experimental approach. Single pulses at three energy densities were applied and the resulting melt pool characteristics were evaluated. The key conclusions are:

- Laser remelting can be used as a technique to eliminate local surface micro-cracks.
- Higher pulse frequency and intensity increase the melt pool temperature and size, while protective gas at 3 bar pressure prevents significant material ablation.
- With higher laser intensity and frequencies, the material hardness increases.
- An increase in intensity and pulse frequency leads to a wrinkled surface on the top layer of the melting pool. The wrinkled surface finally leads to higher surface roughness.
- To achieve a better surface morphology, the minimum input intensity is preferable, as it can improve surface roughness.

As an outlook for practical applications, the findings of this study highlight the potential of the laser remelting process as a post-manufacturing process for titanium biomedical implants. It demonstrates its ability to eliminate micro-cracks and enhance material hardness and morphology.

## Figures and Tables

**Table 1:** Laser parameters.

**Scanning Velocity** **(mm/s)**	**Power** **(W)**	**Frequency** **(Hz)**	**Assis Gas Pres.** **(bar)**	**Pulse Duration** **(ms)**
160	80–160–240	300–500	3	2.6

## Data Availability

The original contributions presented in this study are included in the article. Further inquiries can be directed to the corresponding author.
